# Evolution of Liver Resection for Hepatocellular Carcinoma: Change Point Analysis of Textbook Outcome over Twenty Years

**DOI:** 10.3390/medicina61010012

**Published:** 2024-12-26

**Authors:** Yeshong Park, Ho-Seong Han, Seung Yeon Lim, Hyelim Joo, Jinju Kim, MeeYoung Kang, Boram Lee, Hae Won Lee, Yoo-Seok Yoon, Jai Young Cho

**Affiliations:** Department of Surgery, Seoul National University Bundang Hospital, Seoul National University College of Medicine, Seongnam-si 13620, Gyeonggi-do, Republic of Korea; parkys@snubh.org (Y.P.); hanhs@snubh.org (H.-S.H.); 82891@snubh.org (S.Y.L.); kaylin-j@snubh.org (H.J.); 82750@snubh.org (J.K.); mykang@snubh.org (M.K.); boramlee0827@snubh.org (B.L.); lansh@snubh.org (H.W.L.); yoonys@snubh.org (Y.-S.Y.)

**Keywords:** hepatocellular carcinoma, radiofrequency ablation, salvage hepatectomy, laparoscopic liver resection

## Abstract

*Background and Objectives*: The aim of this study was to comprehensively analyze the evolution in textbook outcome (TO) achievement after liver resection for hepatocellular carcinoma (HCC) over two decades at a single tertiary referral center. *Materials and Methods*: All consecutive liver resections for HCC at Seoul National University Bundang Hospital from 2003 to 2022 were analyzed. The included 1334 patients were divided into four groups by time intervals identified through change point analysis. TO was defined as no intraoperative transfusions, positive margins, major complications, 30-day readmission or mortality, and prolonged length of hospital stay (LOS). *Results*: Multiple change point analysis identified three change points (2006, 2012, 2017), and patients were divided into four groups. More recent time interval groups were associated with older age (59 vs. 59 vs. 61 vs. 63 years, *p* < 0.0001) and more comorbidities. Minimally invasive procedures were increasingly performed (open/laparoscopic/robotic 37.0%/63.0%/0%) vs. 43.8%/56.2%/0% vs. 17.1%/82.4%/0.5% vs. 22.9%/75.9%/1.2%, *p* < 0.0001). TO achievement improved over time (1.9% vs. 18.5% vs. 47.7% vs. 62.5%, *p* < 0.0001), and LOS was the greatest limiting factor. *Conclusions*: TO after liver resection improved with advances in minimally invasive techniques and parenchymal sparing procedures, even in older patients with more comorbidities and advanced tumors.

## 1. Introduction

Conventional quality indicators of surgical care focus on single outcome parameters including postoperative length of hospital stay (LOS), morbidity, and mortality [1,2,3]. Although such single outcome variables are easy to target for quality improvement programs, they often fail to capture the multidimensional aspects of postoperative management. Especially, parameters with low incidence rates such as postoperative mortality have low discrimination power to detect changes in quality of care [4].

Textbook outcome (TO) is a multidimensional composite measure to evaluate the entire surgical care process [5]. TO is achieved only when all parameters are fulfilled, and it is known to represent an ideal postoperative course [6,7]. Generally, TO has higher event rates compared to single outcome variables and provides more power to detect differences between individual hospitals. Change point analysis allows for the identification of changing trends in TO achievement over a certain time sequence in a single institution, which reflects improvement in surgical care associated with specific changes in patient characteristics, operative methods, and postoperative care system.

Liver resection is performed as the standard treatment for benign and malignant liver diseases [8]. Moreover, minimally invasive approach is considered as the reference modality for an increasing range of liver resection procedures [9,10]. TO in liver resection has been evaluated through previous studies with varying definitions [11,12,13,14]. Yet TO achievement rates show great variance across studies, and an expert Delphi consensus has shown that LOS, an important component of TO, is especially affected by differences in cultural aspects and health care systems [15]. For such reasons, using the same definition of TO for interhospital comparison has limited feasibility. Therefore, analysis of consecutive liver resections at a single center performed by the same surgical standards could be more helpful in identifying factors associated with improvement in achieving TO.

The aim of this study is to assess the change in TO achievement rate after liver resection over time in a single-center consecutive cohort collected over twenty years via change point analysis based on the Bayesian information criterion. The parameters to define TO were set according to previous studies. In addition, multivariable regression analysis was performed to evaluate factors associated with achieving TO in liver resection.

## 2. Materials and Methods

### 2.1. Patient Selection

All consecutive patients who underwent liver resection for hepatocellular carcinoma (HCC) between 1 January 2003 and 31 December 2022 at Seoul National University Bundang Hospital were eligible for this study. Inclusion criteria included: (1) histological diagnosis of HCC after surgery; (2) curative intent surgical resection; and (3) available data regarding TO parameters. Patients diagnosed with combined hepatocellular-cholangiocarcinoma or benign hepatic tumors, patients with missing data regarding TO parameters, patients who underwent thermal ablation without surgical resection, and patients for whom the operation was terminated after exploration due to disseminated disease were excluded. Finally, 1334 patients were included for analysis. The study was approved by the institutional review board of each participating institution (IRB No.: B-2211-795-101). This article was prepared in compliance with the Strengthening the Reporting of Observational studies in Epidemiology (STROBE) guidelines for cohort studies [16].

### 2.2. Data Collection and Definitions

Information about patient demographics, operative data, pathological features of the tumor and underlying liver condition, and survival data were collected from the medical records. Hepatectomy procedures were categorized as described by the Brisbane classification [17]. Major liver resection was defined as the resection of three or more adjacent liver segments, such as hemihepatectomy or trisectionectomy. Minor liver resection included non-anatomical liver wedge resection, segmentectomy, or sectionectomy. Anatomical resection was defined as complete removal of the liver parenchyma confined within the responsible portal territory [18]. R0 resection margin was defined as 1 mm or more tumor-free margin. Major complications were defined as complications within 30 days after surgery of Clavien-Dindo grade 3a or higher [19]. 30-day mortality was defined as death during hospitalization or within 30 days after surgery. 30-day readmission was defined as unplanned readmission within 30 days after discharge. Disease-free survival (DFS) was defined as the interval between the date of surgery and the date at which recurrence was first recognized or the date of the last follow-up. Overall survival (OS) was calculated from the date of surgery to the date of death or date of the last follow-up, obtained from either medical records or public administration data.

### 2.3. Textbook Outcomes

Parameters for TO were selected based on previously published definitions and literature describing outcome parameters after liver surgery [11,12,13,14,15]. Factors commonly included in related studies included absence of intraoperative incidents, negative margins, absence of serious postoperative complications including bile leak, absence of postoperative reintervention, no unplanned readmission after discharge, no immediate postoperative mortality after surgery, and no prolonged LOS. Detailed definitions varied between studies, including the estimation period for readmission or postoperative mortality (30 days versus 90 days), cutoff for prolonged LOS (defined as ≤50th, ≤ 75th, and ≤90th percentile), and inclusion of specific complications as independent TO items (bile leakage or liver failure).

The final criteria for TO achievement in the current study utilized parameters overlapping between previously proposed definitions, which included no intraoperative transfusions, negative margins, absence of major complications, absence of 30-day mortality, absence of 30-day readmission, and no prolonged LOS. LOS is a dynamic factor affected not only by the functional recovery of the patient, but also by cultural differences, healthcare systems, and funding mechanisms. For this reason, definitions for prolonged LOS differ between individual studies, and the International Expert Delphi Consensus on Defining Textbook Outcome in Liver Surgery has proposed different cutoffs for LOS according to regions (Europe/Africa/Middle East, Americans, and Asian Pacific) [15]. In the current study, the cut-off value of 50th percentile of the total cohort was calculated and used as the definition of prolonged LOS as in a previous study from our institution [14].

### 2.4. Multiple Change Point Analysis

**Figure 1:**
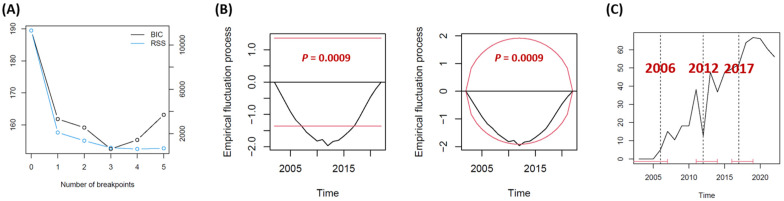
Multiple change point analysis was performed to identify distinct change points in textbook outcome achievement. (**A**) Comparison of Bayesian Information Criterion (BIC) values for different numbers of change points revealed that three change points were optimal. (**B**) The number of breakpoints was confirmed as statistically significant based on ordinary least-squares-based cumulative sum test. (**C**) Three change points were identified at year 2006, 2012, and 2017.

To precisely identify the time intervals when significant changes in TO achievement had occurred, multiple change point analysis was performed. Change points were estimated using the ‘strucchange’ package in R Project for Statistical Computing (version 4.3.3; R Foundation for Statistical Computing, Vienna, Austria), which allows for simultaneous estimation of multiple breakpoints in time series regression models [20,21,22] The ‘breakpoints’ function was applied to estimate the number and location of change points. This method identifies all potential change points in the time series data by comparing Bayesian Information Criterion (BIC) values for different numbers of change points, and the model having the lowest BIC value is considered optimal (Figure 1A). The number of breakpoints was further confirmed as statistically significant based on ordinary least-squares-based cumulative sum test (*p* = 0.0009; Figure 1B). Finally, three change points (2006, 2012, 2017) were identified (Figure 1C).

**Figure 2:**
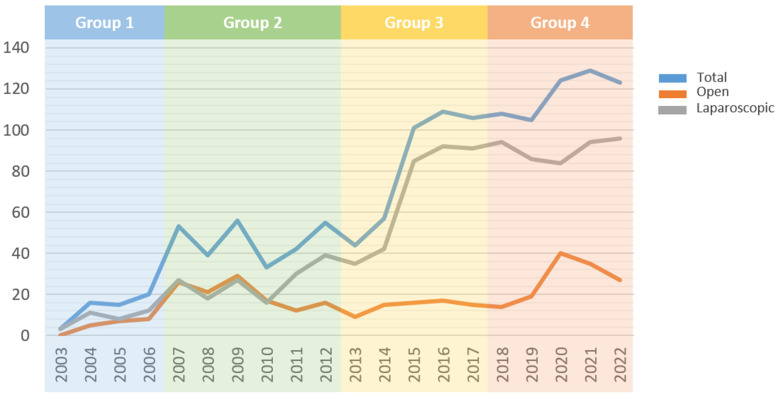
Trends in number of annual operations during each time interval.

Based on the change points, patients were divided into four time interval groups: group A (1 January 2003~31 December 2006; *n* = 54), group B (1 January 2007~31 December 2012; *n* = 276), group C (1 January 2013~31 December 2017; *n* = 415) and group D (1 January 2018~31 December 2022; *n* = 589). An increase in number of operations performed was noted over time, especially in minimally invasive operations (Figure 2). Baseline clinicopathological characteristics, perioperative outcomes, long-term oncologic outcomes, and TO achievement were compared between groups.

### 2.5. Statistical Analysis

Descriptive statistics were used to compare clinicopathological characteristics and TO between the time interval groups. Categorical variables were presented as number (percentage) and compared using chi-square test or Fisher’s exact test. Normally distributed continuous variables were presented as mean ± standard deviation and analyzed using the one-way analysis of variance (ANOVA). Non-normally distributed continuous variables were presented as median (interquartile range) and analysed with Kruskal-Wallis rank sum test. Survival analysis was conducted using the Kaplan‒Meier method with the log-rank test. Univariable and multivariable Cox regression analyses were performed to identify factors associated with TO achievement. All *p*-values were two-sided, and *p* < 0.05 was considered statistically significant. Statistical analyses were performed using R version 4.3.3 Project for Statistical Computing.

## 3. Results

### 3.1. Baseline Characteristics

The changes in clinicopathological characteristics across time intervals are shown in Table 1. More recent time interval groups were associated with older age (group A vs. B vs. C vs. D: 59 (52–68) vs. 59 (51–67) vs. 61 (52–69) vs. 63 (56–70) years, *p* < 0.0001), higher body mass index (group A vs. B vs. C vs. D: 24.4 ± 3.3 vs. 23.7 ± 3.2 vs. 24.3 ± 3.4 vs. 25.1 ± 3.3 kg/m^2^, *p* < 0.0001), and higher proportion of underlying disease including hypertension, diabetes mellitus, dyslipidemia, and cardiovascular disease. Alcohol consumption (group A vs. B vs. C vs. D: 10 (18.5%) vs. 108 (39.1%) vs. 142 (34.2%) vs. 312 (53.0%), *p* < 0.0001) increased over time. The liver function of operated patients showed improvement in the more recent time intervals, with a higher proportion of Child Pugh A patients (group A vs. B vs. C vs. D: 46 (85.2%) vs. 264 (95.7%) vs. 395 (95.2%) vs. 581 (98.6%), *p* < 0.0001) and lower MELD score (group A vs. B vs. C vs. D: 7.8 (7.2–9.7) vs. 7.7 (7.0–8.9) vs. 7.5 (6.9–8.2) vs. 6.9 (6.4–7.8), *p* < 0.0001).

### 3.2. Operative Parameters and Postoperative Outcomes

When operative parameters were compared, group C and group D were associated with a higher proportion of minimally invasive procedures (group A vs. B vs. C vs. D: open/laparoscopic/robotic 20/34/0 (37.0%/63.0%/0%) vs. 121/155/0 (43.8%/56.2%/0%) vs. 71/342/2 (17.1%/82.4%/0.5%) vs. 135/447/7 (22.9%/75.9%/1.2%), *p* < 0.0001; Table 2). More recent time intervals were associated with higher proportion of minor resection (group A vs. B vs. C vs. D: 38 (70.4%) vs. 194 (70.3%) vs. 345 (83.1%) vs. 486 (82.5%), *p* < 0.0001) and non-anatomical resection (group A vs. B vs. C vs. D: 21 (38.9%) vs. 93 (33.7%) vs. 345 (83.1%) vs. 486 (82.5%), *p* < 0.0001). LOS also gradually decreased over time (group A vs. B vs. C vs. D: 11 (8–18) vs. 9 (7–12) vs. 6 (5–8) vs. 5 (4–7), *p* < 0.0001).

### 3.3. Pathologic Features

Pathological features according to different time intervals were analyzed (Table 3). More recent time interval groups showed larger number of tumors (group A vs. B vs. C vs. D: 1 (1–3) vs. 1 (1–5) vs. 1 (1–5) vs. 1 (1–13), *p* = 0.034) and higher Edmonson-Steiner grade (grade IV in group A vs. B vs. C vs. D: 3 (5.6%) vs. 29 (10.8%) vs. 54 (13.5%) vs. 158 (27.7%), *p* < 0.0001). The proportion of patients with liver cirrhosis decreased over time (group A vs. B vs. C vs. D: 29 (53.7%) vs. 173 (62.7%) vs. 245 (59.0%) vs. 185 (31.4%), *p* < 0.0001).

### 3.4. Achievement of TO

**Figure 3:**
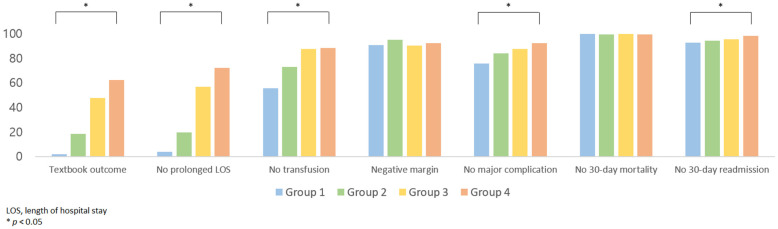
Achievement of overall textbook outcome and its individual components over time.

The overall TO achievement rate of the entire cohort was 43.2%. TO achievement rate improved over time (group A vs. B vs. C vs. D: 1.9% vs. 18.5% vs. 47.7% vs. 62.5%, *p* < 0.0001; Figure 3). When individual items were analyzed, significant improvement was noted in no intraoperative transfusions (group A vs. B vs. C vs. D: 55.6% vs. 73.2% vs. 87.7% vs. 88.5%, *p* < 0.0001), absence of major complications (group A vs. B vs. C vs. D: 75.9% vs. 84.1% vs. 87.5% vs. 92.4%, *p* < 0.0001), absence of 30-day readmission (group A vs. B vs. C vs. D: 92.6% vs. 94.2% vs. 95.4% vs. 98.5%, *p* = 0.0009), and no prolonged LOS (group A vs. B vs. C vs. D: 3.7% vs. 19.6% vs. 56.9% vs. 72.3%, *p* < 0.0001). Negative margins (group A vs. B vs. C vs. D: 90.7% vs. 95.3% vs. 90.4% vs. 92.2%, *p* = 0.291) and absence of 30-day mortality (group A vs. B vs. C vs. D: 100% vs. 99.3% vs. 99.8% vs. 99.5%, *p* = 0.677) showed no change over time.

Prolonged LOS was the greatest limiting factor in achieving TO. When the cutoff for prolonged LOS was changed to the 75th and 90th percentile, overall TO achievement rate increased to 63.0% and 68.9%, respectively.

### 3.5. Multivariable Analysis for TO Achievement

Univariable and multivariable Cox regression analyses were performed for factors associated with TO achievement (Table 4). In the univariable analysis, older age (hazard ratio [HR] 0.75, *p* = 0.032), higher Child Pugh score (HR 0.29, *p* < 0.001), major liver resection (HR 0.34, *p* < 0.001), higher T stage (T3: HR 0.29, *p* < 0.001, T4: HR 0.31, *p* < 0.001) and presence of liver cirrhosis (HR 0.71, *p* = 0.002) were negative predictors for TO achievement. Laparoscopic (HR 5.36, *p* < 0.001) and robotic approaches (HR 14.90, *p* < 0.001) were associated with improved TO. In multivariable analysis, older age (HR 0.71, *p* = 0.015), higher Child Pugh score (HR 0.41, *p* = 0.020), major liver resection (HR 0.57, *p* = 0.001), presence of liver cirrhosis (HR 0.64, *p* < 0.001), and minimally invasive approach (laparoscopic: HR 4.30, *p* < 0.001; robotic: HR 11.20, *p* = 0.003) remained significant.

## 4. Discussion

Surgical resection is the only potentially curable option for patients with HCC and is therefore considered the treatment of choice in patients with good hepatic function [8]. Although surgical indications have been extended to larger tumors and more difficult locations during the past decades, liver resection remains a challenging procedure with high risk of perioperative morbidity [23]. A recent global, multicenter prospective study reported that 42% of hepatectomy patients experienced postoperative complication, with a 16% major complication rate [24]. For such reasons, standardized measurement of surgical quality and identification of specific factors to target for improvement are important to further refine the quality of care in HCC patients. TO is currently considered as an ideal composite measure to evaluate postoperative outcomes in various types of operations, and achievement of TO is also actively evaluated for liver resection [25,26,27,28].

In the current study, we analyzed consecutive liver resections performed at a single center over twenty years for TO achievement. Overall, 43.2% of all patients achieved TO, with patients operated in the most recent time interval reaching a TO achievement rate of 62.5%. It should be noted that the definition for TO utilized in the current study was more strictly set compared to previous studies, with the cutoff for prolonged LOS defined at 50th percentile. Previous studies reported TO achievement rate of approximately 60% defining prolonged LOS by the 75th or 90th percentile; we also reported similar achievement rates of 63.0% and 68.9% with extended definitions for prolonged LOS [11,12,13]. Therefore, the overall TO achievement rate in the current study was comparable to existing literature.

Older patients with more comorbidities were increasingly operated over time, together with an increase in the number and grade of tumors. This is in line with previous studies which showed that patients with higher risk of morbidity were increasingly offered a chance of surgical resection due to extended resection criteria [23,29,30]. With advances in surgical techniques and improvement in patient management, elderly patients have shown short-term and long-term outcomes comparable to younger age groups, although concerns regarding decreased liver regeneration capacity still exist [31]. The indication for resection has also been extended to single large tumors with more aggressive behavior, which is also reflected in the results of the current study [32,33].

Hepatic function of patients eligible for surgery showed improvement over time in the current study; on the contrary, an increasing Albumin-Bilirubin (ALBI) score was reported in a previous study on TO achievement over time [23]. In South Korea, where the most common cause of HCC is chronic hepatitis B virus infection, high risk patients are offered active prevention and intensive surveillance programs for HCC by the national healthcare system [34,35]. Due to advances in antiviral treatment and successful management of liver cirrhosis, a study based on the Korean Nationwide Cancer Registry has reported that the proportion of surgical candidates with preserved liver function is gradually increasing over time [36]. The results of the current study reflect such cultural backgrounds. In addition, effective surveillance with ultrasonography and the combined use of alpha-fetoprotein and protein induced by vitamin K absence II (PIVKA-II) was actively implemented in 2007 in our center; the subsequent increase in early detection of HCC could have contributed to the first change point in TO achievement [37].

More operations were conducted via minimally invasive approaches over time, with minor and non-anatomical resections being increasingly performed. Technical advances in laparoscopic and robotic liver resection have led to less postoperative pain, lower postoperative morbidity, and shorter LOS [9,10,38]. In the current study, minimally invasive approach was a significant predictor for TO achievement. The annual surgical volume significantly increased with the expansion of minimally invasive surgery, which also correlated with change points in TO achievement. Expanded use of minimally invasive Pringle maneuver, more sophisticated parenchymal dissection techniques, and laparoscopic or robotic devices such as staplers, ultrasonic dissectors, and hydrodissectors might have contributed to specific change points [39,40,41]. The evolving field of laparoscopic and robotic liver resection is expected to lead to higher TO achievement rates in the future. Results from the current study are promising in that surgeons could further improve patient outcomes through technical advances including image-guided surgery, real-time navigation, and the robotic platform.

Kingham et al. analyzed liver resections performed at Memorial Sloan Kettering over twenty years and reported that morbidity and mortality rates substantially dropped as major hepatectomies decreased [42]. The results of the current study confirmed such trends, which suggests that there has been a general shift in surgical technique towards parenchymal preservation that has also contributed to improved surgical outcomes [43]. The oncologic outcomes after anatomical and non-anatomical resection remain controversial. Several studies have suggested that anatomical resection offers benefits in securing resection margin and removing micrometastases [44,45,46]. On the other hand, non-anatomical resection with adequate surgical margins shows benefits in shorter operation time, less estimated blood loss, and lower transfusion rate, with oncologic outcomes comparable to anatomical resection [47,48]. In the current study, improvement in TO achievement was noted despite increase in non-anatomical resections over time; this also supports the notion that non-anatomical resection could offer postoperative benefits to patients if margins are adequately secured.

Certain limitations should be considered in interpreting the results of the current study. First, the study was limited by its retrospective nature. Second, the study period covered consecutive liver resections performed over twenty years, and there could have been substantial changes in the operative principles and techniques for liver resection over time. To reduce the confounding effect by these factors, change point analysis was adapted. Lastly, the current study utilized the 50th percentile as the definition for prolonged LOS and 30-day rather than 90-day readmission and mortality rates. Detailed definitions for TO vary across studies, and such differences might impact the comparative analysis of results.

## 5. Conclusions

In conclusion, TO achievement showed gradual improvement over time, with distinct change points being identified. Change points correlated with older patient age, more comorbidities, improved liver function, larger tumor size, higher tumor grade, more minor or non-anatomical resections, and increase in minimally invasive surgery. These trends suggest that patients who were traditionally deemed unresectable were increasingly offered a chance for surgical treatment with advances in minimally invasive techniques and parenchymal sparing procedures.

## Figures and Tables

**Table 1:** Baseline demographics and clinical characteristics of time interval groups.

	Group 1 (*n* = 54)	Group 2 (*n* = 276)	Group 3 (*n* = 415)	Group 4 (*n* = 589)	*p*-Value
Age (years)	59 (52–68)	59 (51–67)	61 (52–69)	63 (56–70)	*<0.0001*
Sex (male:female)	40:14	211:65	342:73	476:113	0.163
Body mass index (kg/m^2^, mean ± SD)	24.4 ± 3.3	23.7 ± 3.2	24.3 ± 3.4	25.1 ± 3.3	*<0.0001*
Underlying disease					
Hypertension	8 (14.8)	99 (35.9)	163 (39.3)	310 (52.6)	*<0.0001*
Diabetes mellitus	2 (3.7)	68 (24.6)	113 (27.2)	186 (31.6)	*<0.0001*
Dyslipidemia	2 (3.7)	10 (3.6)	33 (8.0)	88 (14.9)	*<0.0001*
Heart disease	1 (1.9)	7 (2.5)	25 (6.0)	59 (10.0)	*0.0001*
Habits					
Alcohol	10 (18.5)	108 (39.1)	142 (34.2)	312 (53.0)	*<0.0001*
Smoking	8 (14.8)	49 (17.8)	112 (27.0)	361 (61.3)	*<0.0001*
Child Pugh score					*<0.0001*
A	46 (85.2)	264 (95.7)	395 (95.2)	581 (98.6)	
B	8 (14.8)	12 (4.3)	20 (4.8)	8 (1.4)	
MELD score	7.8 (7.2–9.7)	7.7 (7.0–8.9)	7.5 (6.9–8.2)	6.9 (6.4–7.8)	*<0.0001*

Values are median (interquartile range) or n (%) unless otherwise indicated. SD, standard deviation; MELD, Model for End-Stage Liver Disease. The significant *p*-Values are marked in italics.

**Table 2:** Operative parameters and postoperative outcomes.

	Group 1 (*n* = 54)	Group 2 (*n* = 276)	Group 3 (*n* = 415)	Group 4 (*n* = 589)	*p*-Value
Operative method					*<0.0001*
Open	20 (37.0)	121 (43.8)	71 (17.1)	135 (22.9)	
Laparoscopic	34 (63.0)	155 (56.2)	342 (82.4)	447 (75.9)	
Robotic	0	0	2 (0.5)	7 (1.2)	
Resection extent					*<0.0001*
Minor resection	38 (70.4)	194 (70.3)	345 (83.1)	486 (82.5)	
Major resection	16 (29.6)	82 (29.7)	70 (16.9)	103 (17.5)	
Anatomical resection	33 (61.1)	183 (66.3)	210 (55.6)	260 (44.1)	*<0.0001*
Tumor location					0.665
S1–5	32 (59.3)	167 (60.5)	247 (59.5)	333 (56.5)	
S6–8	22 (40.7)	109 (39.5)	168 (40.5)	256 (43.5)	
Operative time	300 (220–418)	280 (190–360)	205 (145–300)	145 (95–210)	*<0.0001*
Estimated blood loss	800 (500–1475)	500 (300–1000)	350 (150–700)	300 (100–600)	*<0.0001*
Transfusion	24 (44.4)	74 (26.8)	51 (12.3)	68 (11.5)	*<0.0001*
Complication	19 (35.2)	63 (22.8)	90 (21.7)	252 (42.8)	*<0.0001*
≥Clavien-Dindo grade IIIa	13 (24.1)	44 (15.9)	52 (12.5)	45 (7.6)	*<0.0001*
Length of hospital stay (days)	11 (8–18)	9 (7–12)	6 (5–8)	5 (4–7)	*<0.0001*
30-day readmission	4 (7.4)	16 (5.8)	19 (4.6)	9 (1.5)	*0.0009*
30-day mortality	0	2 (0.7)	1 (0.2)	3 (0.5)	0.677

Values are median (interquartile range) or n (%) unless otherwise indicated. The significant *p*-Values are marked in italics.

**Table 3:** Pathological features of time interval groups.

	Group 1 (*n* = 54)	Group 2 (*n* = 276)	Group 3 (*n* = 415)	Group 4 (*n* = 589)	*p*-Value
Size	4.2 ± 3.3	4.0 ± 2.7	3.8 ± 3.1	3.8 ± 3.0	0.254
Number	1 (1–3)	1 (1–5)	1 (1–5)	1 (1–13)	*0.034*
T stage					
T1	26 (48.1)	135 (48.9)	194 (46.7)	278 (47.2)	*<0.0001*
T2	14 (25.9)	92 (33.3)	162 (39.0)	254 (43.1)	
T3	5 (9.3)	32 (11.6)	27 (6.5)	27 (4.6)	
T4	2 (3.7)	6 (2.2)	25 (6.0)	23 (3.9)	
Surgical margin					0.291
R0	49 (90.7)	263 (95.3)	375 (90.4)	543 (92.2)	
R1	4 (7.4)	12 (4.3)	36 (8.7)	38 (6.5)	
Cirrhosis	29 (53.7)	173 (62.7)	245 (59.0)	185 (31.4)	*<0.0001*
Edmonson-Steiner grade (worst)					*<0.0001*
1	1 (1.9)	3 (1.1)	6 (1.5)	3 (0.5)	
2	13 (24.1)	75 (27.9)	93 (23.3)	89 (15.6)	
3	30 (55.6)	151 (56.1)	241 (60.3)	315 (55.3)	
4	3 (5.6)	29 (10.8)	54 (13.5)	158 (27.7)	

Values are median (interquartile range) or *n* (%) unless otherwise indicated. The significant *p*-Values are marked in italics.

**Table 4:** Cox regression analyses for TO achievement.

	Univariable		Multivariable	
	HR (95% CI)	*p*-Value	HR (95% CI)	*p*-Value
Age				
<70	Ref.		Ref.	
≥70	0.75 (0.58–0.98)	*0.032*	0.71 (0.53–0.93)	*0.015*
Body mass index				
<25	Ref.		Ref.	
≥25	1.34 (1.08–1.67)	*0.009*	1.19 (0.94–1.51)	0.150
Child-Pugh score				
A	Ref.		Ref.	
B	0.29 (0.14–0.57)	*<0.001*	0.41 (0.18–0.84)	*0.020*
Operation method				
Open	Ref.		Ref.	
Laparoscopic	5.36 (4.01–7.25)	*<0.001*	4.30 (3.15–5.94)	*<0.001*
Robotic	14.90 (3.51–102.00)	*<0.001*	11.20 (2.58–76.80)	*0.003*
Resection extent				
Minor	Ref.		Ref.	
Major	0.34 (0.25–0.46)	*<0.001*	0.57 (0.41–0.80)	*0.001*
Tumor location				
S1-5	Ref.			
S6-8	1.00 (0.80–1.24)	0.999		
Cirrhosis				
No	Ref.		Ref.	
Yes	0.71 (0.57–0.88)	*0.002*	0.64 (0.50–0.81)	*<0.001*
pT				
T1	Ref.		Ref.	
T2	0.81 (0.64–1.02)	0.079	1.00 (0.78–1.28)	0.999
T3	0.29 (0.17–0.48)	*<0.001*	0.62 (0.35–1.08)	0.100
T4	0.31 (0.16–0.56)	*<0.001*	0.71 (0.35–1.38)	0.300
Edmonson-Steiner grade				
1	Ref.			
2	1.67 (0.53–6.28)	0.400		
3	2.03 (0.66–7.56)	0.200		
4	2.44 (0.77–9.21)	0.150		

HR, hazard ratio; CI, confidence interval. The significant *p*-Values are marked in italics.

## Data Availability

The data presented in this study are available on request from the corresponding author.
